# Exercise Improves Metabolism and Alleviates Atherosclerosis via Muscle-Derived Extracellular Vesicles

**DOI:** 10.14336/AD.2022.1131

**Published:** 2023-06-01

**Authors:** Yixiao Wang, Yunnan Liu, Siyan Zhang, Na Li, Changyang Xing, Chen Wang, Jia Wang, Mengying Wei, Guodong Yang, Lijun Yuan

**Affiliations:** ^1^Department of Ultrasound Diagnostics, Tangdu Hospital, Fourth Military Medical University, Xi'an, China.; ^2^Department of Ultrasound Diagnostics, Jintai Hospital, Baoji, China.; ^3^The State Laboratory of Cancer Biology, Department of Biochemistry and Molecular Biology, Fourth Military Medical University, Xi'an, China.

**Keywords:** extracellular vesicles, exercise, metabolism, atherosclerosis, skeletal muscle

## Abstract

Regular exercise maintains a healthy metabolic profile, while the underlying mechanisms have not been fully elucidated. Extracellular vesicles serve as an important mediator in intercellular communication. In this study, we aimed to explore whether exercise-induced extracellular vesicles (EVs) of skeletal muscle origins contribute to exercise-related protective effects on metabolism. We found that the twelve weeks of swimming training improved glucose tolerance, reduced visceral lipid accumulation, alleviated liver damage, and inhibited atherosclerosis progression in both obese WT mice and ApoE^-/-^ mice, which could be partially blocked by EV biogenesis repression. Injection of skeletal muscle-derived EVs from exercised C57BL/6J mice (twice a week for 12 weeks) had similar protective effects on both obese WT mice and ApoE^-/-^ mice as exercise itself. Mechanistically, these exe-EVs could be endocytosed by major metabolic organs, especially the liver and adipose tissue. With the protein cargos rich in mitochondrial and fatty acid oxidation-related components, exe-EVs remodeled metabolism towards beneficial cardiovascular outcomes. Our study here has shown that exercise remodels metabolism towards beneficial cardiovascular outcomes at least partially via the skeletal muscle secreted EVs. Therapeutic delivery of exe-EVs or the analogues could be promising for prevention of certain cardiovascular and metabolic diseases.

## INTRODUCTION

There is an increasing worldwide burden of cardiometabolic disorders, such as hyperlipidemia, obesity, and insulin resistance. Cardiometabolic disorders are the common risk factors for atherosclerosis (AS) [1-4], which is characterized by excessive lipid deposition in the arterial wall [5]. AS is the pathological basis of cardiovascular disease (CVD) and is one of the leading causes of morbidity and mortality worldwide [6, 7]. Regular physical exercise can effectively improve metabolism, delay the development of AS, and thus reduce CVD mortality [8-10]. However, the detailed mechanisms by which physical exercise could protect the subject against cardiovascular diseases remains largely unknown.

Besides energy expenditure, physical exercise also changes myokines which have systemic functions. Recently, extracellular vesicles (EVs), secreted by nearly all cell types of the body, are found to play a critical role in intercellular communication via transfer the information from the donor cells to the recipient cells [11, 12]. EV has been found to be closely associated with cardiovascular health [13]. Pioneering studies have also shown that EV has therapeutic potential in protecting the heart from ischemic injury [14]. Moreover, EV has been used as a potential biomarker for CVD [15]. Studies have shown that exercise not only changes the number of EVs but also the cargos encapsulated [16, 17], and thus the circulating exosome pools are also altered [18]. However, whether exercise-related EVs of skeletal muscle origin involved in cardiometabolic improvement are largely unknown.

In the present study, we found that skeletal muscle derived EVs from exercised mice are enriched with proteins involved in mitochondrial biogenesis and fatty acid β-oxidation. These EVs in turn could be up-taken by metabolic organs, such as liver and adipose tissues, reshaping metabolic disorders and alleviating AS. Our study not only reveals a novel mechanism by which exercise improves metabolism, but also provides a new putative intervention strategy for AS prevention.

## MATERIALS AND METHODS

### Animal experiments

Male C57BL/6J 8-week-old mice were obtained from the Experimental Animal Center of Fourth Military Medical University. Male ApoE^-/-^ 8-week-old mice on a C57BL/6 background were obtained from Model Animal Research Center of Nanjing University. All animal experiments were approved by the Animal Care and Use Committee of Fourth Military Medical University, in accordance with the National Institutes of Health Guide for the Care and Use of Laboratory Animals (NIH Publication No. 85-23). All mice were housed in specific pathogen-free conditions at 22°C-24°C and 40-60% humidity, with a 12 light/12 dark cycle. After seven days of adaptation, C57BL/6J mice were fed a normal chow diet and were subjected to a 4-week exercise protocol to obtain exercise related muscle extracellular vesicles. Obese C57BL/6 mice were induced by feeding with a high-fat diet for 12 weeks. In order to induce atherosclerosis, ApoE^-/-^ mice were fed with a high-fat diet for 12 weeks (D12492, study diet containing 45% fat, 20% protein, and 20% carbohydrate). At the end of the experiment, mice were anesthetized with 120 mg/kg body weight of ketamine and 24 mg/kg body weight of xylazine, and then sacrificed by CO_2_ exposure.

### Exercise protocol and additional treatment

The swim training protocol was modified from previous studies [19]. Briefly, an endurance swimming program was undertaken five times a week in the exercise group in a temperature-controlled water bath (35-36 °C). All training sessions were conducted from 5:00 p.m. to 6:00 p.m. Before the formal exercise program begins, mice have a 5-minute swimming session to acclimate. The endurance swimming program began with 10 minutes of swimming once a day and increased by 5 minutes every day to 30-minute sessions each day.

For extracellular vesicles biogenesis inhibition, GW4869 (S7609, Selleck, USA) dissolved in DMSO (D8418, Sigma, USA) was injected intraperitoneally at a single dose of 2.5 μg/g three times per week [20].

### Blood tests

For blood glucose tolerance test, mice were intraperitoneally injected with glucose at 2 g/kg, and then blood glucose from tail vein was measured at 0, 15, 30, 60, and 120 minutes after injection utilizing an ACCU-CHEK glucometer (Roche, Germany). For other blood tests, the whole blood was collected from the eyeballs of anesthetized mice. The blood was centrifuged for 3,000 g at 4 °C after being placed at room temperature for 20 minutes, and the supernatant was collected for further analysis. Total cholesterol (TC), total triglyceride (TG), high-density lipoprotein (HDL) cholesterol, and low-density lipoprotein (LDL) cholesterol, liver function test including the measurement of ALT (Alanine Amino-transferase) and AST (Aspartate Aminotransferase) were measured using Chemray 240 and Chemray 800 Chemistry analyzers (Rayto, Shenzhen, China).

### Noninvasive pulse wave velocity (PWV) measurements

Mouse PWVs were measured by experienced technicians using Vevo 2100 Imaging System (FUJIFILM, VisualSonics, Canada) armed with MS550 transducer (22-55 MHz). A chemical hair remover was used to remove mouse hairs in the chest and abdomen. After anesthesia with isoflurane (2% induction, 1.2% maintenance), the mice were placed on a heating pad with temperature control to maintain their body temperature. During the examination, the heart rate was maintained between 400-450 beats per minute. Peak velocities of the ascending and abdominal aortic arteries were measured using PWV Doppler mode. PWV was calculated by the following formula: D/[T2-T1] (m/s), in which T1 is the starting time of R wave in the ascending aortic Doppler waveform, and T2 is the starting time of R wave in the abdominal aortic Doppler waveform. D is the distance between the ascending aorta and the abdominal aorta [21]. A spectrum analyzer and real-time signal acquisition system were used to measure the parameters.

### Extracellular vesicles isolation, characterization, and tail vein injection

For isolation of muscle-derived EV, muscles were collected from sacrificed mice under sterile conditions, followed by cut into small pieces less than 1mm^3^ and cultured in serum-free DMEM for 24 h. The culture medium was centrifuged at 300 g for 10 minutes, and 10,000 g for 30 minutes to remove the cell debris and/or dead cells. The sample was then ultracentrifuged at 100,000 g for 70 minutes, and the pellet was resuspended in sterile PBS and ultracentrifuged for another 70 minutes at 100,000 g. The pellets were resuspended in PBS at a suitable concentration and stored at -80°C till use.

To isolate plasma EVs, mice were anesthetized and then the whole blood was collected as described above and stored in anticoagulated tubes containing EDTA. EV isolation was performed using commercial kit ExoQuick™ (ExoQ5TM-1/TMEXO-1, SBI, USA) as instructed.

**Figure 1.**
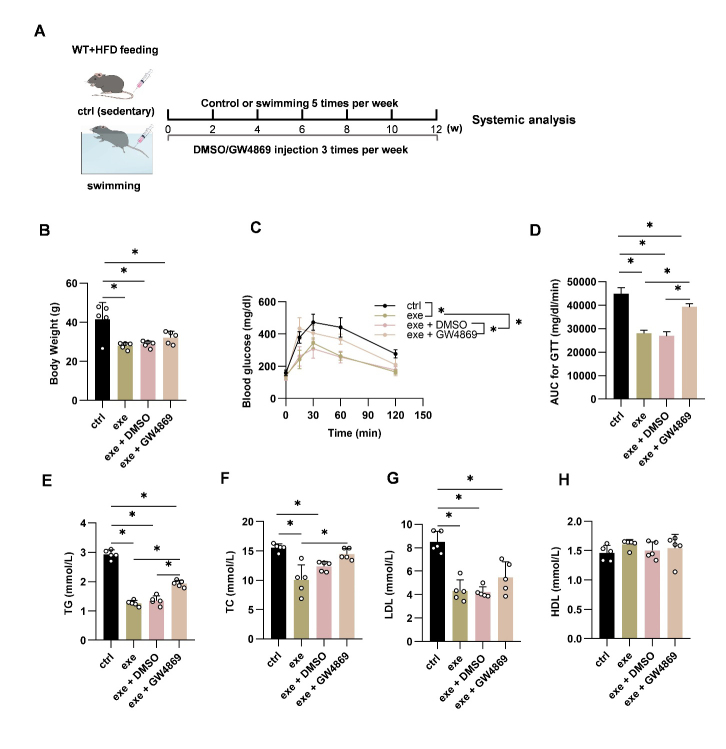
EV biogenesis inhibition reverses the beneficial metabolic effects of exercise in WT mice. (A) Illustration of animal grouping and experimental procedure. (B) Body weights of the mice with indicated treatments. (C) Intraperitoneal glucose tolerance test in the mice with indicated treatments. (D) AUC of GTT in the mice with indicated treatments. E-H. Blood lipid analysis. Serum total triglycerides (E), total cholesterol (F), low-density lipoprotein cholesterol (G), and high-density lipoprotein cholesterol (H) from mice of indicated groups. Data are presented as mean ± SEM. *, *P*<0.05; Kruskal-Wallis test with Dunn's post hoc test. n=5 per group. AUC, area under the curve; GTT, glucose tolerance test; TG, total triglyceride; TC, total cholesterol; LDL, low-density lipoprotein; HDL, high-density lipoprotein.

EV size distribution and concentration were determined by NanoSight NS300 (Malvern, Egham, UK). Transmission electron microscopy (JEOL, Tokyo, Japan) was used to observe the morphology of extracellular vesicles. Exosome inclusive markers CD9 and TSG101, and exclusive marker GM130 were used to confirm the identity of isolated EVs. The specific procedures were the same as previously described [22]. In the study, primary antibodies included anti-CD9 (ab29726, Abcam), anti-TSG101 (ab125011, Abcam), anti-GM130 (ab30637, Abcam), and anti-GAPDH (60004-1-Ig, Proteintech). Horseradish peroxidase-conjugated goat anti-rabbit (7074, CST) and goat anti-mouse (7076, CST) secondary antibodies were used in the study.

For in vivo and ex vivo tracking of EV, mice were injected with 100 μg DiR/DiI labeled EVs via tail vein and sacrificed 4 hours later for IVIS or fluorescence microscopic analysis. For EV treatment, mice were injected with 100 μg indicated EVs via tail vein twice a week for 12 weeks, followed by systemic analysis.

### Histology

ApoE^-/-^ mice were intracardially perfused with ice-cold phosphate-buffered saline (PBS) and 4% para-formaldehyde sequentially after deep anesthesia. Then, the aortas were exposed, and the surrounding adipose tissues were removed, with the aortic arch macroscopic view taken by a digital camera. The en-face aorta was stained with Oil Red O, and the percentage lesion area was calculated using Image J. Hematoxylin and eosin (H&E) staining, Oil Red O staining, and Masson's trichome staining were performed in the aortic root cross section to identify atherosclerotic areas, lipid deposition, and collagen content. In addition, H&E and Oil Red O staining were also performed on liver sections. eWAT sections were also included for H&E staining, adipocyte areas and diameters were calculated.

### Extracellular vesicle labeling and in vivo tracing

For tracking the distribution of EVs *in vivo*, purified EVs were incubated with 8 μM DiR or DiI (Invitrogen, Waltham, USA) at 37 °C for 30 min, and free DiR or DiI was removed by another round of EV isolation. About 100 μg DiR-labeled EVs were injected into the mice via tail vein and IVIS Lumina II system (PerkinElmer, Thermo Fisher, USA) was used to measure EV distribution among the main organs 4 hours after injection. To further profile cellular distribution of EV in the liver, heart, spleen, and other tissues, mice were injected with DiI-labeled EVs and the organs were removed four hours after injection, followed by embedded in optimum cutting temperature (OCT) compound (Sakura, Finetek, USA) and sectioned. DAPI (Invitrogen, Waltham, USA) was used to counterstain nuclei. Fluorescence signal was detected and imaged by confocal laser scanning microscope (Nikon A1R, Tokyo, Japan).

### Mass Spectrometry

EVs isolated from muscles of control or exercised mice were resuspended in PBS. RIPA lysis buffer was used to extract the proteins from the EVs and total proteins from EVs were separated by electrophorese. Then, the gel was subjected to mass spectrometry. Mass spectrometry and bioinformatics analysis were performed by Bangfei Bioscience (China). An Orbitrap Fusion Lumos (Thermo Scientific, USA) was used to analyze trypsinized samples separated by high performance liquid chromatography (HPLC). GO enrichment and KEGG pathway enrichment were used to identify differentially expressed proteins.

### Statistical analysis

Data are presented as mean ± SEM. The Shapiro-Wilk test was used to determine the normality of the data distribution. If distributions were normal, student t-test was used for the comparison of two groups. If distributions were nonnormal or N was too small (N<6), a Mann-Whitney U-test was used for the comparison of two groups, and Kruskal-Wallis test with Dunn’s post hoc test for multiple-group comparisons. P<0.05 was considered statistically significant for comparisons between groups using post hoc analysis (GraphPad Prism 8.0).

## RESULTS

### Swim Training improves the metabolism in obese mice via EVs

It was shown that exercise has induced robust changes of EVs [16]. To explore whether EVs are involved in exercise-mediated metabolic remodeling, we first used GW4869 (a widely used blocker of exosome biogenesis and secretion [23-25]) to block the biogenesis and secretion of EV. As expected, injection with GW4869 in WT mice resulted in a significant decrease in plasma EV concentration (Supplementary Fig. 1A-C). In the following experiments, HFD fed WT mice were subjected to a 12-week swimming regimen and additionally injected with control or GW4869 intraperitoneally three times a week 4 h before exercise (Fig. 1A). HFD induced body weight increase was significantly inhibited in swimming-trained mice (exe), while this benefit was not affected by GW4869 (Fig. 1B). In contrast, exe-mice increased glucose tolerance as evidenced by the lower area under the curve (AUC) of glucose tolerance test (GTT). Additional treatment of GW4869 suppressed the effects (Fig. 1C, D). Exercise also reduced TG, TC, and LDL cholesterol, and GW4869 treatment blunted the protective effects of exercise on TG and TC. GW4869 treatment had no significant effects on LDL and HDL (Fig. 1E-H). Interestingly, GW4869 treatment on sedentary control mice had no significant effects on the metabolic parameters examined (Supplementary Fig. 2A-H). Overall, the above results demonstrate that exercise ameliorates metabolic disorders in obese mice and GW4869 at least partially attenuates exercise-mediated metabolic benefits by inhibiting EV biogenesis.

**Figure 2.**
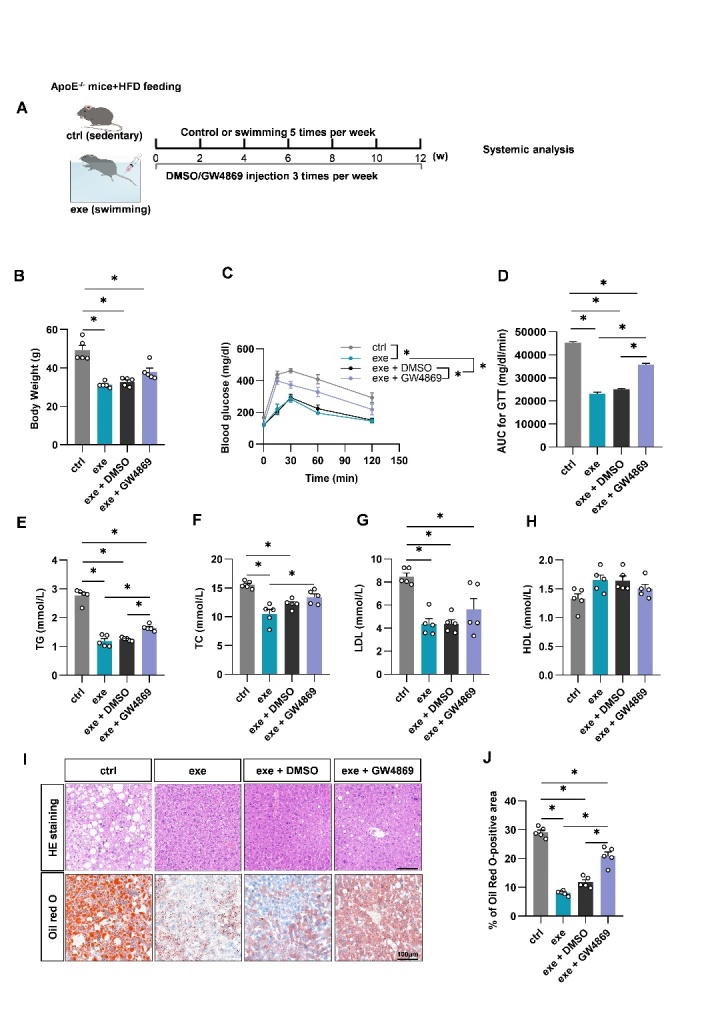
EV biogenesis inhibition reverses the beneficial metabolic effects of exercise in ApoE^-/-^ mice. (A) Illustration of animal grouping and experimental procedures. (B) Body weights of the mice with indicated treatments. (C) Intraperitoneal glucose tolerance test in the mice with indicated treatments. (D) AUC of GTT in the mice with indicated treatments. (E-H) Blood lipid analysis. Serum total triglycerides (E), total cholesterol (F), low-density lipoprotein cholesterol (G), and high-density lipoprotein cholesterol (H) from mice of indicated groups. (I) HE staining (top) and oil red O staining (bottom) of liver sections from indicated groups. (J) Percentage of Oil Red O positive area in livers from indicated groups. Data are presented as mean ± SEM. *, *P*<0.05; Kruskal-Wallis test with Dunn's post hoc test. n=5 per group.

**Figure 3.**
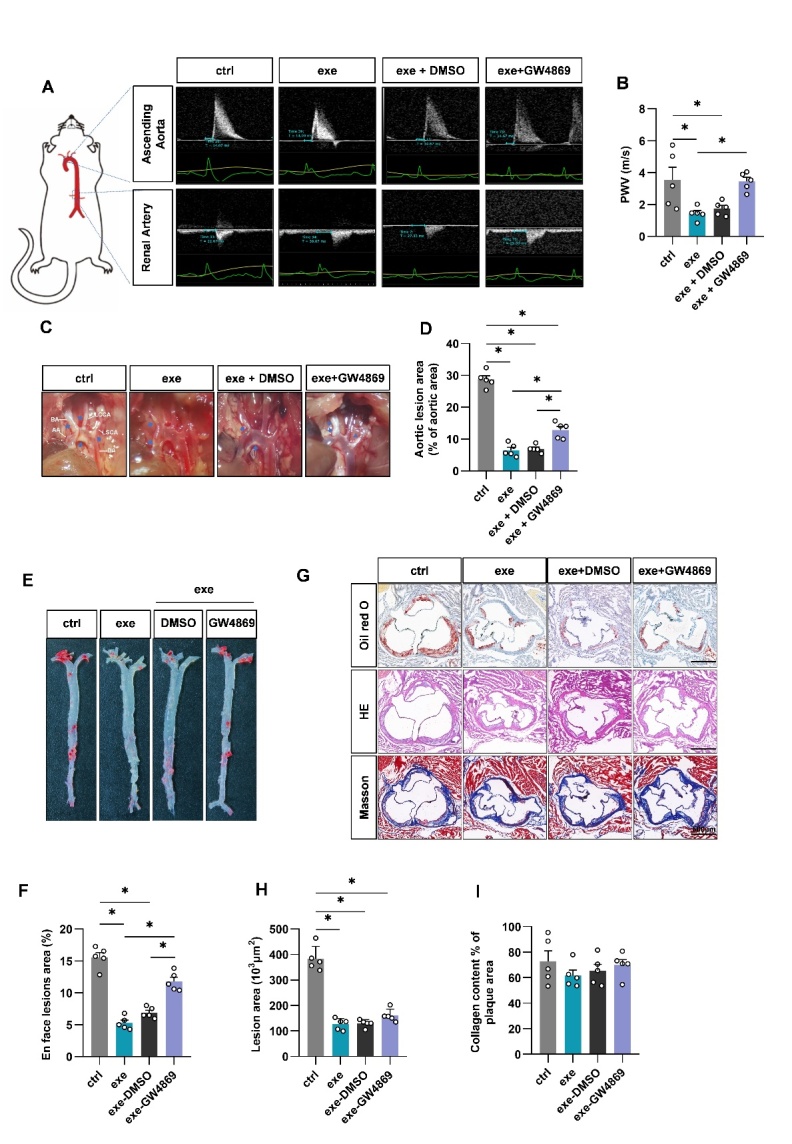
Swimming training alleviates atherosclerosis in ApoE^-/-^ mice via EVs. (A) Cartoons showing how PWV was acquired and representative images of the ultrasound examination. (B) PWV values from mice with indicated treatments. (C) Representative aortic arch view of the atherosclerotic lesions in indicated mice. (D) Percentage of the atherosclerotic area in the aortic arch. (E) Oil red O staining of the aortic tree from indicated mice. (F) Percentage analysis of the atherosclerotic region corresponding to Panel E. (G) Representative images of the cross-sectional view of the aortic roots stained with Oil red O, HE and Masson. (H-I) Percentage analysis of the atherosclerotic lesion area (H) and collagen content corresponding to Panel F. Data are presented as mean ± SEM. *, *P*<0.05; Kruskal-Wallis test with Dunn's post hoc test. n=5 per group. PWV, pulse wave velocity; AA, ascending aorta; BA, brachiocephalic artery; LCCA, left common carotid artery; LSCA, left subclavian artery; DA, descending aorta.

**Figure 4.**
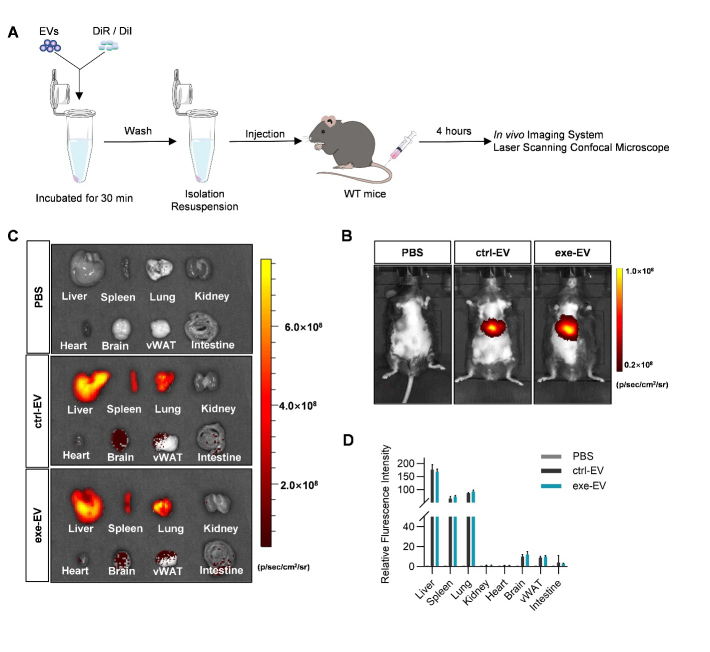
*In vivo* distribution of muscle derived EVs in WT mice. (A) Illustration of experimental procedure. (B) Representative images of the *in vivo* distribution of DiR-labeled EVs were obtained by an *in vivo* imaging system. (C) *Ex vivo* fluorescence imaging analysis of the distribution of the DiR-labeled EVs in different organs. (D) Quantification of fluorescence Intensity in C. n=5 per group.

### Swim Training alleviates atherosclerosis in ApoE^-/-^ mice via EVs

Given the causal role of systemic metabolic disorders in atherosclerosis, we next explored whether swim training alleviated atherosclerosis in an EV dependent manner in ApoE^-/-^ mice (Fig. 2A). GW4869 treatment also decreased plasma EV concentration in ApoE^-/-^ mice (Supplementary Fig. 3A-C). Similarly in obese WT mice, swimming training improved the metabolic profile of ApoE^-/-^ mice (Fig. 2B-H), and the protective effects on glucose tolerance, TG, and TC were at least partially blocked by GW4869 (Fig. 2C-F). In addition, exercise also reduced lipid accumulation in hepatocytes of ApoE^-/-^ mice, while additional treatment of GW4869 partially suppressed the effects (Fig. 2I, J). Similarly, exercise reduced the size of visceral adipocytes, which could be slightly inhibited by GW4869 (Supplementary Fig. 4A-C). To evaluate hepatic function, we also tested serum glutamic aminotransferase (ALT) and glutamic oxalacetic aminotransferase (AST), and the results showed that exercise significantly decreased AST and ALT in ApoE^-/-^ mice, while additional treatment of GW4869 attenuated the protective effects (Supplementary Fig. 4D, E). Notably, GW4869 treatment on sedentary ApoE^-/-^ mice had no significant effects on the metabolic parameters examined (Supplementary Fig. 5A-O). In conclusion, EVs at least partially contribute to the exercise-mediated metabolic remodeling.

**Figure 5.**
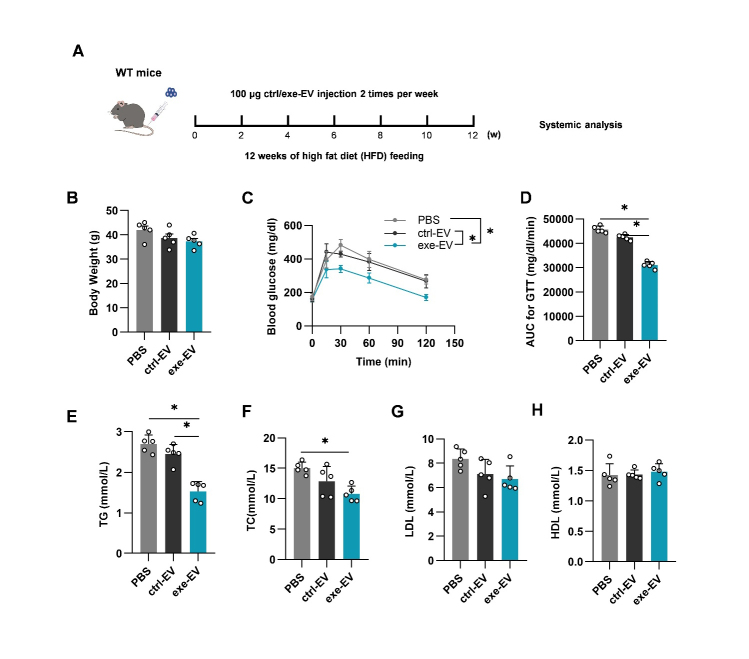
Exe-EV treatment improves the metabolic profile in WT mice. (A) Illustration of animal grouping and experimental procedure. B. Body weights of the mice receiving PBS, ctrl-EV or exe-EV treatments. (C) Intraperitoneal glucose tolerance test in the mice with indicated treatments. (D) AUC of GTT in the mice with indicated treatments. E-H. Serum lipid levels from indicated groups. Serum total triglycerides (E), total cholesterol (F), low-density lipoprotein cholesterol (G), and high-density lipoprotein cholesterol (H) from mice of indicated groups. Data are presented as mean ± SEM. *, *P*<0.05; Kruskal-Wallis test with Dunn's post hoc test. n = 5 per group.

Consistent with the beneficial effects on metabolism, exercise also delayed progression of atherosclerosis in EV dependent manner. We first assessed arterial stiffness by PWV, a classical indicator of vascular stiffness [26]. Compared to the control group, the aortic PWV of exercised ApoE^-/-^ mice was much lower. Additional treatment of GW4869 partially blocked the PWV decrease (Fig. 3A, B). Accordingly, exercise reduced the plaque size and number in aorta of ApoE^-/-^ mice. Notably, Masson staining showed that the collagen content in the aortic roots did not change in exercise group (Fig. 3G, I). GW4869 treatment reduced the protective effects of exercise (Fig. 3C-H), while GW4869 alone had minimal effects on atherogenesis (Supplementary Fig. 6). Together, these data revealed that exercise alleviates atherosclerosis in ApoE^-/-^ mice at least partially via EVs.

### Exe-EVs remodels systemic metabolism similar as exercise in obese WT mice

Given the data described above, we hypothesized that muscle-derived EVs might be involved in the process. EVs from muscle tissue of control (ctrl-EV) and exercised mice (exe-EV) were thus isolated (Supplementary Fig. 7A). Nanoparticle tracking analysis (NTA) showed no significant difference in particle size distribution, with the concentration slightly increased in exercised group (Supplementary Fig. 7B, C). Transmission electron microscopy (TEM) analysis showed that both ctrl-EV and exe-EV were typically round in shape and ranged 80 ~ 100 nm in diameter (Supplementary Fig. 7D). Western blot analysis clarified the presence of exosome marker proteins (TSG101, CD9) and absence of exclusive marker protein GM130 [27], confirming that ctrl-EV and exe-EV are mainly exosomes (Supplementary Fig. 7E, with the original western blot bands showing in Supplementary Fig. 8).

**Figure 6.**
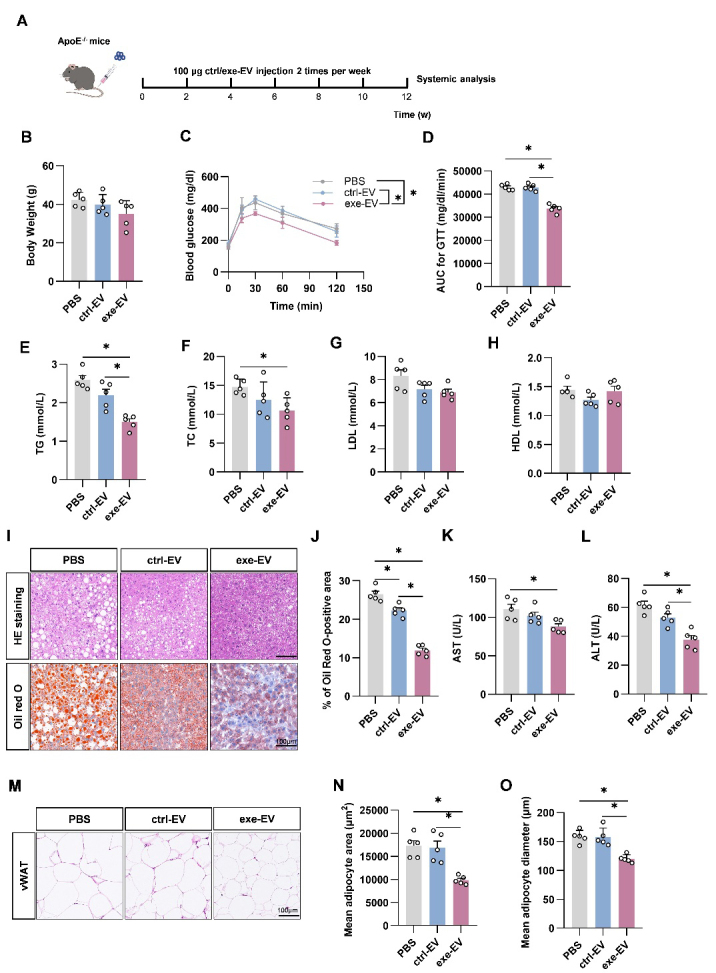
Exe-EV treatment improves the metabolic profile and hepatic function in ApoE^-/-^ mice. (A) Illustration of animal grouping and experimental procedure. (B) Body weights of ApoE^-/-^ mice receiving PBS, ctrl-EV and exe-EV treatments. (C) Intraperitoneal glucose tolerance test in the mice with indicated treatments. (D) AUC of GTT in the the mice with indicated treatments. (E-H) Serum lipid levels from indicated groups. Serum total triglycerides (E), total cholesterol (F), low-density lipoprotein cholesterol (G), and high-density lipoprotein cholesterol (H) from mice of indicated groups. (J) Percentage of Oil Red O positive area in livers from indicated groups. (K-L) Serum levels of AST (K) and ALT (L) in mice from each group. (M) HE staining of vWAT from indicated mice. N. Quantification of mean adipocyte area of vWAT. O. Quantification of mean adipocyte diameters of vWAT. Data are presented as mean ± SEM. *, *P*<0.05; Kruskal-Wallis test with Dunn's post hoc test. n = 5 per group.

**Figure 7.**
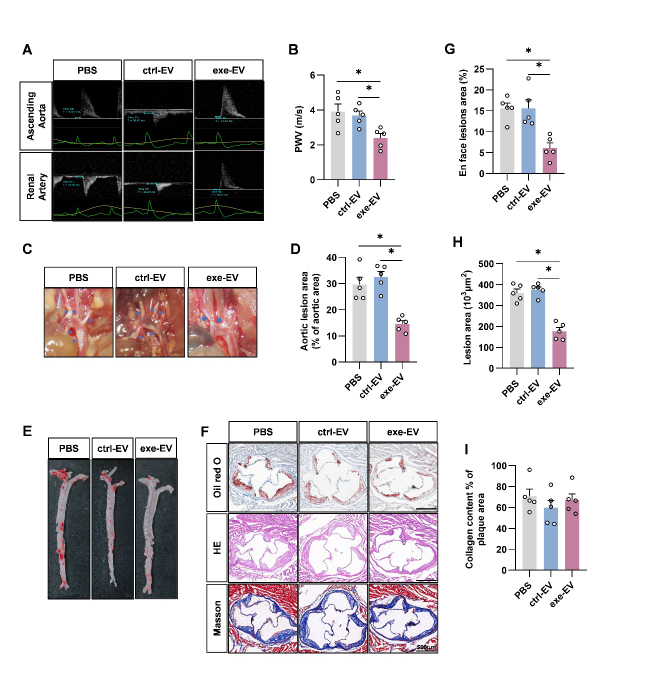
Exe-EV treatment alleviates atherosclerosis in ApoE^-/-^ mice. (A) Representative images showing the examination of PWV. (B) PWV in ApoE^-/-^ mice treated as indicated. (C) Representative aortic arch view of the atherosclerotic lesions in in ApoE^-/-^ mice treated as indicated. (D) Percentage of the atherosclerotic area in the aortic arch. (E) Representative images showing oil red O staining of the aortic tree in ApoE^-/-^ mice treated as indicated. (F) Representative images of the cross-sectional view of the aortic roots stained with oil red O, HE and Masson. (G) Percentage analysis of the atherosclerotic region from E. (H-I) Percentage analysis of the atherosclerotic lesional area (H) and collagen content corresponding to Panel F. Data are presented as mean ± SEM. *, *P*<0.05; Kruskal-Wallis test with Dunn's post hoc test. n = 5 per group.

To explore whether exe-EV has protective effects similar to exercise, we first tracked DiR/DiI-labeled muscle EVs (Fig. 4A). Both DiR-labeled ctrl-EV and exe-EV were found mainly transported to the liver, spleen, and lung in WT mice. Besides, ctrl-EV and exe-EV were also sparsely present in some other organs, such as the visceral WAT (Fig. 4B-D). The distribution profile of EVs was further confirmed by laser confocal microscopy analysis of DiI-labeled ctrl-EV and exe-EV (Supplementary Fig. 9A, B).

We next injected WT mice fed with high-fat diet with 100 µg ctrl-EV or exe-EV by tail vein twice a week for 12 weeks (Fig. 5A). No differences in body weight were observed among the different groups (Fig. 5B). However, exe-EV treatment improved glucose tolerance (Fig. 5C, D), and reduced TG (Fig. 5E) and TC levels (Fig. 5F), while had no significant effects on LDL and HDL levels (Fig. 5G, H). Overall, exe-EV could improve the metabolism profile in obese WT mice.

### Exe-EVs alleviates atherosclerosis in ApoE^-/-^ mice similar as exercise

In view of the above data, we next aimed to investigate the possibility of exe-EVs to alleviate atherosclerosis. The same exe-EVs treatment protocol was conducted in ApoE^-/-^ mice (Fig. 6A). As expected, improved glucose tolerance and decreased TG and TC were found in the exe-EVs treated group (Fig. 6C-F). In contrast, body weight, LDL, and HDL did not change significantly in mice with exe-EVs treatment (Fig. 6B, G, H).

Exe-EV had a similar distribution profile in ApoE^-/-^ mice as in WT mice, with liver and spleen as the dominant organs (Supplementary Fig. 10, S11), suggesting that liver might be one of the key effector organs in EV mediated cardiometabolic protection. To further explore the therapeutic effects of EVs, 100 µg of ctrl-EV and exe-EV were injected into ApoE^-/-^ mice twice a week for 12 weeks via tail vein. Similar to that observed in exercised ApoE^-/-^ mice, exe-EVs treatment reduced the lipid deposition in the liver (Fig. 6I, J). In addition, exe-EV treatment significantly reduced the AST and ALT (Fig. 6K, L). Moreover, there was also a significant reduction of adipocyte size in visceral adipose tissue in mice with exe-EV treatment (Fig. 6M-O). In contrast, no such protective effects were observed in ApoE^-/-^ mice receiving ctrl-EV treatment.

We next examined whether exe-EV treatment alleviated atherosclerotic lesions. In comparison with the PBS control and ctrl-EV treated groups, exe-EV treatment significantly reduced aortic PWV (Fig. 7A, B), decreased atherosclerotic plaque size and number (Fig. 7C, D). The plaque burden, in particular the lipid core, as examined by H&E staining and oil red O staining, was significantly lowered by treatment of exe-EVs (Fig. 7E-H). However, there were no differences in collagen content in the plaques among the groups (Fig. 7F, I). The above data clearly demonstrated that exe-EV treatment attenuated atherosclerosis in ApoE^-/-^ mice.

### Profiling of the protein components of exe-EVs

In order to identify the bioactive molecules in EVs that might be responsible for exercise-induced metabolic effects, we thus analyzed the protein contents of ctrl-EV and exe-EV by mass spectrometry. A total of 1297 proteins in ctrl-EV and 1311 proteins in exe-EV were identified (Supplementary Fig. 12A). A threshold of fold change ≥2, P<0.05 was used for screening of differential proteins. Compared with ctrl-EV, 192 proteins were upregulated, while 171 proteins were downregulated in exe-EV (Supplementary Fig. 12B-C). Gene Ontology enrichment analysis revealed that upregulated proteins in exe-EV were enriched in fatty acid metabolism and mitochondria biogenesis (Supplementary Fig. 12D). KEGG pathway analysis also revealed that upregulated proteins in exe-EV are mainly involved in carbon metabolism, tricarboxylic acid cycle propanoate metabolism, fatty acid metabolism, and pyruvate metabolism pathways (Supplementary Table 1), which may at least partially explain the metabolic benefits of exe-EV. Future study to reveal the essential component(s) for the beneficial effects might shed light on exe-EV mimetic engineering. It is also important to mention that there might be dozens of proteins together exerting the beneficial function, rather than one or two specific molecule(s).

## DISCUSSION

In the present study, we demonstrated that regular exercise improved glucose tolerance, reduced visceral lipid accumulation, improved liver function, and inhibited the progression of atherosclerosis in murine models, while these systemic benefits could be partially blocked by exosome biogenesis inhibition. Therapeutic delivery of skeletal muscle-derived extracellular vesicles from exercised mice (exe-EVs) could remodel the metabolism in major metabolic organs, particularly the liver and adipose tissue, improving cardiovascular metabolism similar to exercise. Notably, these EVs were enriched in proteins involved mitochondrial function and fatty acid oxidation, providing the molecular basis for their metabolic benefits.

Most cells secrete EVs, which can be absorbed by neighboring cells or distant cells [28]. Previously, adipose-derived EVs were found to be involved in the regulation of metabolism [29, 30]. It is also found exercise related muscle-derived exosomes improve insulin sensitivity by downregulating hepatic FoxO1 in mice [18]. Consistent with the findings that muscle-derived extracellular vesicles regulate the function of liver and adipose tissue [31, 32], we here found that exe-EV could circulate into liver and adipose tissue. Notably, a small fraction also circulated to the heart and aorta, suggesting that some exe-EV can directly affect and regulate the heart and aorta. Exercise has been found to change the EV biogenesis in both yield and cargos [33]. Consistent with the cargo change upon exercise, our proteomic results suggested that mitochondria biogenesis and fatty acid oxidation associated proteins, such as, Suclg1, Sdha, Sdhb, Acly, Idh3b, and Dlat, are enriched in the exe-EVs. It has been shown that there was an increase in the number of exosomes during exercise [34]. However, we didn’t observe a similar effect, possibly due to differences in exercise type or sample collection timing.

It has been well established that exercise has increased energy expenditure and thus benefits metabolism [35]. Remarkably, our data showed that the effects of exercise (body weight loss, LDL decrease) on WT and ApoE^-/-^ mice were not completely inhibited by blocking EV. Previous studies have revealed that exercise could change caloric expenditure [36] and hemodynamic changes [37]. In response to exercise, skeletal muscle also produces, secretes, and releases myokines that act in autocrine, paracrine, and endocrine ways [38, 39]. These myokines related to exercise also have profound beneficial effects [40-42]. Theoretically, decrease in body weight is mainly regulated by energy expenditure. The effect of EVs blockade on lipid metabolism was relatively small compared with that of glucose metabolism, suggesting EV might preferentially regulate glucose metabolism. In other words, energy expenditure, hemodynamic changes, myokines and EVs work in concert to improve metabolism.

It is important to note that myokines have a short half-life in circulation and the function should be transient. EVs contain many bioactive substances, including DNA [43], RNA [44] and proteins [45]. As encapsulated into the vesicles, these bioactive molecules are protected from degradation and thus have longer circulation time and thus sustained function. Although exercise-derived muscle EVs have shown excellent performance in improving cardiovascular metabolism, their sources are very limited. Thus, development of EV mimetics based on decoding the functional components could be of great importance. Further clarifying the detailed molecules responsible for the beneficial effects will shed light on how to produce the exe-EV mimetics. Notably, exe-EVs are actually a heterogeneous population, and it is technically difficult to decode the proteins at single EV level. In addition, all these proteins might exert protective effects in concert, and it is currently impossible to identify which protein plays an essential or fundamental role over others. Besides the protein components, the miRNA components could also not be able to be excluded. To this end, we prefer to engineer the donor cells towards exercise-like muscle cells, rather than encapsulation of specific cargos to the EVs for manufacturing exe-EV mimetics.

Cardiometabolic aberration and atherosclerosis are closely related to aging and aging-associated diseases. Regular physical activity helps maintain a healthy cardiometabolic profile in humans [46, 47-52]. However, physical activity is restricted in certain fragile elderly populations. Therefore, therapeutic delivery of EV mimetics resembling the function of exercise may protect the aging subjects with physical disability from metabolic deterioration, which is an essential goal in a progressive aging society.

## Conclusions

In conclusion, we have demonstrated for the first time that the cardiometabolic benefits generated by regular exercise can be partially blocked by inhibition of EV biogenesis. Exercise-related skeletal muscle-derived extracellular vesicles (exe-EVs) are enriched in mitochondrial function and fatty acid oxidization could transfer the protective cargos to recipient cells, mimicking the effects of exercise. Ongoing studies to develop exercise-derived muscle EV mimics may open new paradigms for the treatment of cardiometabolic diseases, especially in the aged subjects with exercise disability.

## Supplementary Materials

The Supplementary data can be found online at: www.aginganddisease.org/EN/10.14336/AD.2022.1131.
