# Supplementary material for: Exercise Improves Metabolism and Alleviates Atherosclerosis via Muscle-Derived Extracellular Vesicles
Source: Aging Dis. 2023 Jun 1;14(3):952–65. doi: 10.14336/AD.2022.1131 (PMC10187707; doi:10.14336/AD.2022.1131)
Supplement: Supplementary file 1 — The Supplementary data can be found online at: www.aginganddisease.org/EN/10.14336/AD.2022.1131. [file AD-14-3-952-s.pdf]

SUPPLEMENTARY DATA

Exercise Improves Metabolism and Alleviates Atherosclerosis via Muscle-Derived Extracellular Vesicles

Yixiao Wang[1#], Yunnan Liu[1#], Siyan Zhang,[1#], Na Li[1, 2], Changyang Xing[1], Chen Wang[1], Jia Wang[1], Mengying Wei[3*], Guodong Yang[3*], Lijun Yuan[1*]

© 2022. Wang Y et al. Published online at http://www.aginganddisease.org/EN/10.14336/AD.2022.1131

SUPPLEMENTARY DATA

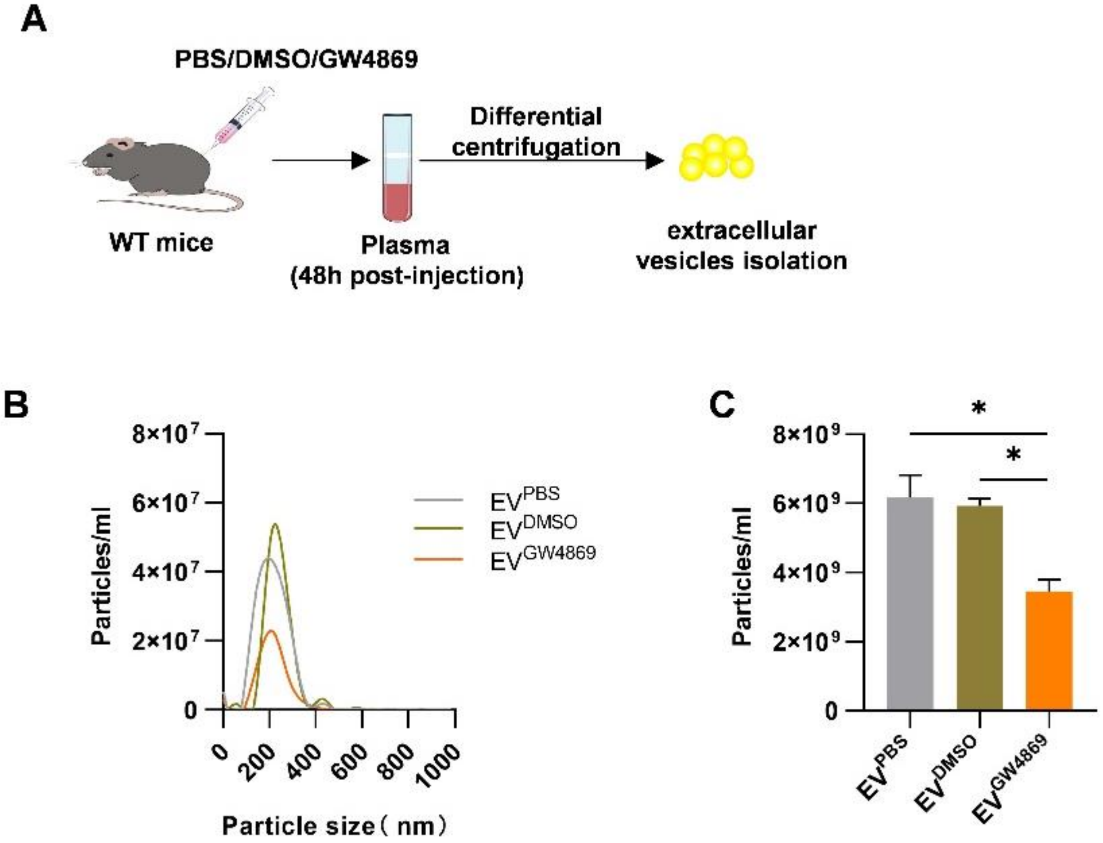

Supplementary Figure 1. GW4869 blocks EV secretion in WT mice. A. Illustration of experimental procedure. B. Representative nanoparticle tracking analysis demonstrating the size distribution of purified plasma EVs from mice injected with or without GW4869. C. Concentration of plasma EVs from mice injected with or without GW4869. Data are presented as mean ± SEM. *, $P<0.05$; Kruskal-Wallis test with Dunn's post hoc test. n = 5 per group.

© 2022. Wang Y et al. Published online at http://www.aginganddisease.org/EN/10.14336/AD.2022.1131

SUPPLEMENTARY DATA

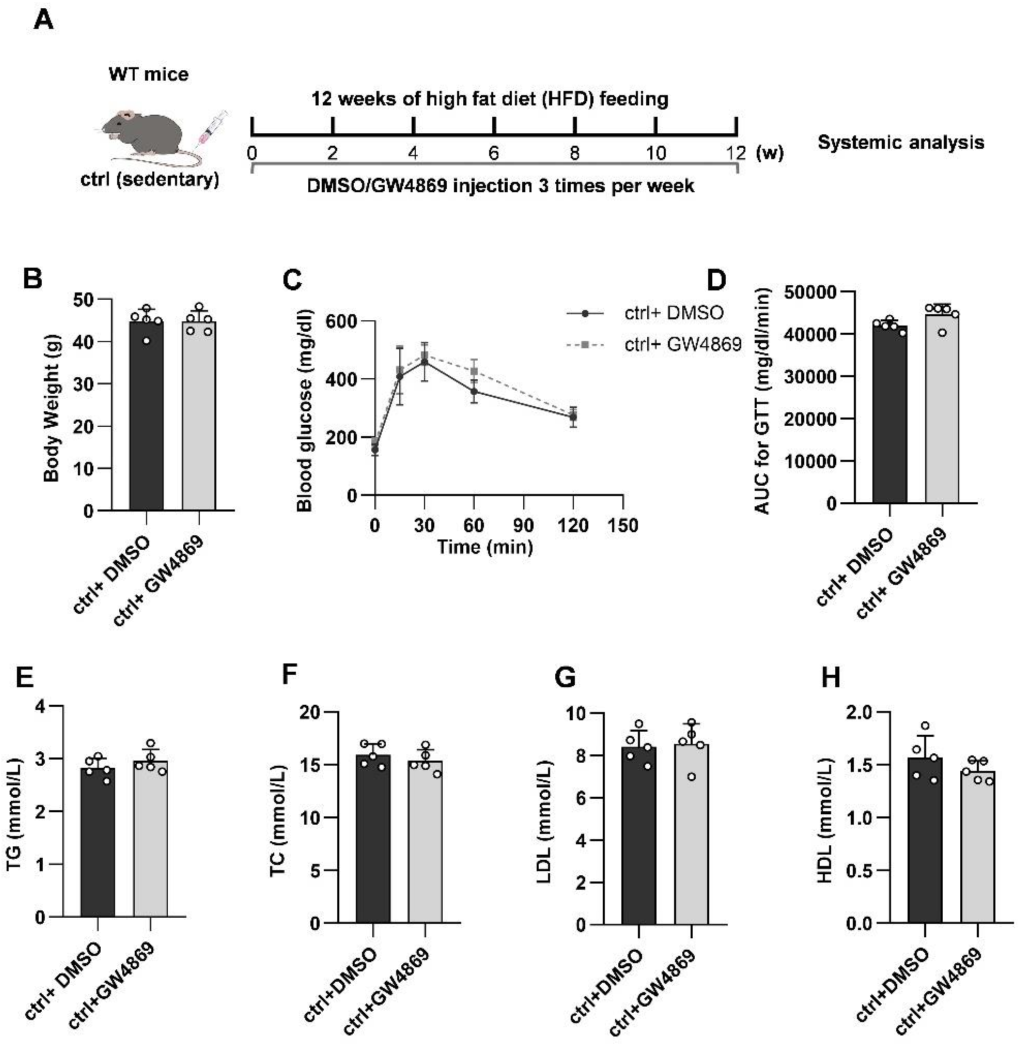

Supplementary Figure 2. GW4869 has no effects on metabolic profile in non-exercised WT mice. A. Illustration of animal grouping and experimental procedure. B. Body weights of the WT mice injected with or without GW4869. C. Intraperitoneal glucose tolerance test in WT mice injected with or without GW4869. D. AUC of the GTT in WT mice injected with or without GW4869. E-H. Blood lipid analysis. Serum total triglycerides (E), total cholesterol (F), low-density lipoprotein cholesterol (G), and high-density lipoprotein cholesterol (H) from mice of indicated groups. Data are presented as mean ± SEM. *, P<0.05; two-tailed Mann-Whitney U test. n = 5 per group.

© 2022. Wang Y et al. Published online at http://www.aginganddisease.org/EN/10.14336/AD.2022.1131

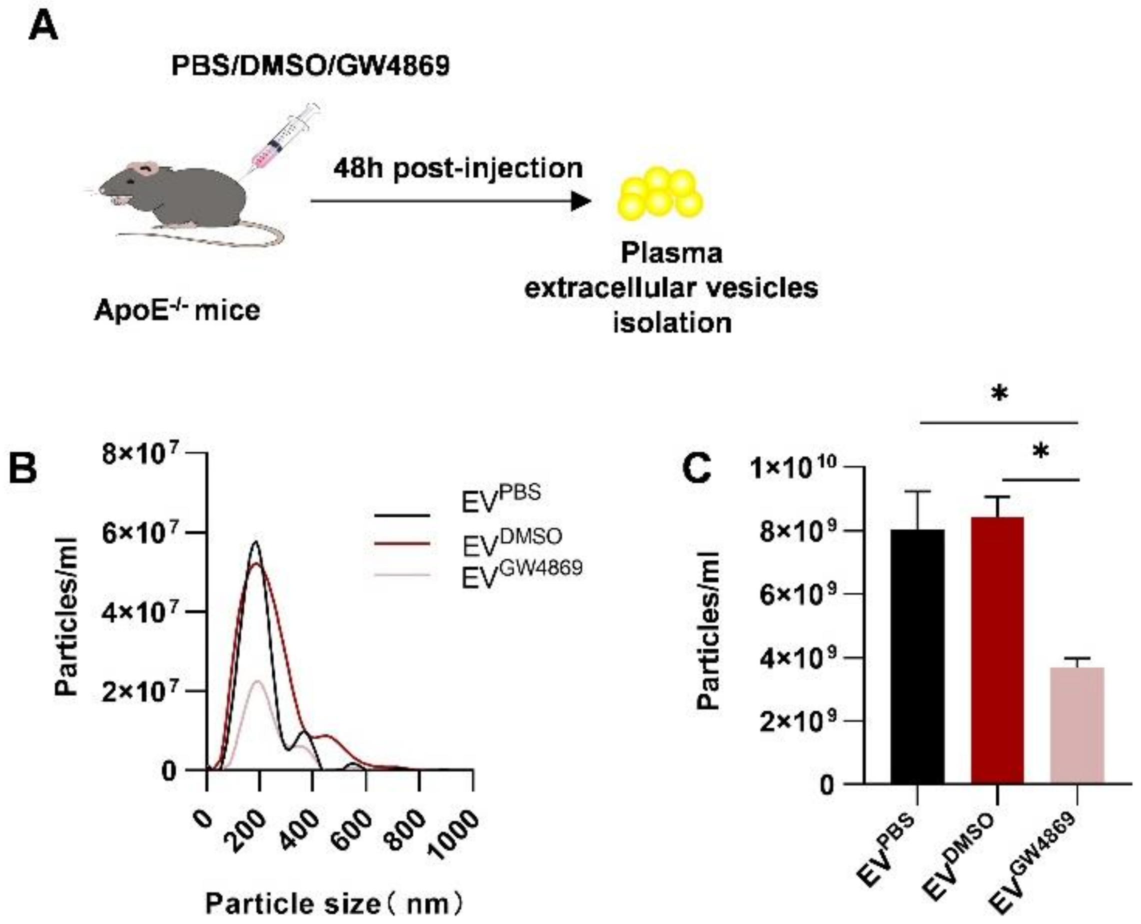

Supplementary Figure 3. GW4869 blocks EV secretion in ApoE$^{-/-}$ mice. A. Illustration of experimental procedure. B. Representative nanoparticle tracking analysis demonstrating the size distribution of purified plasma EVs from ApoE$^{-/-}$ mice injected with or without GW4869. C. Concentration of plasma EVs from mice injected with or without GW4869. Data are presented as mean ± SEM. *, $P<0.05$; Kruskal-Wallis test with Dunn's post hoc test. n = 5 per group.

© 2022. Wang Y et al. Published online at http://www.aginganddisease.org/EN/10.14336/AD.2022.1131

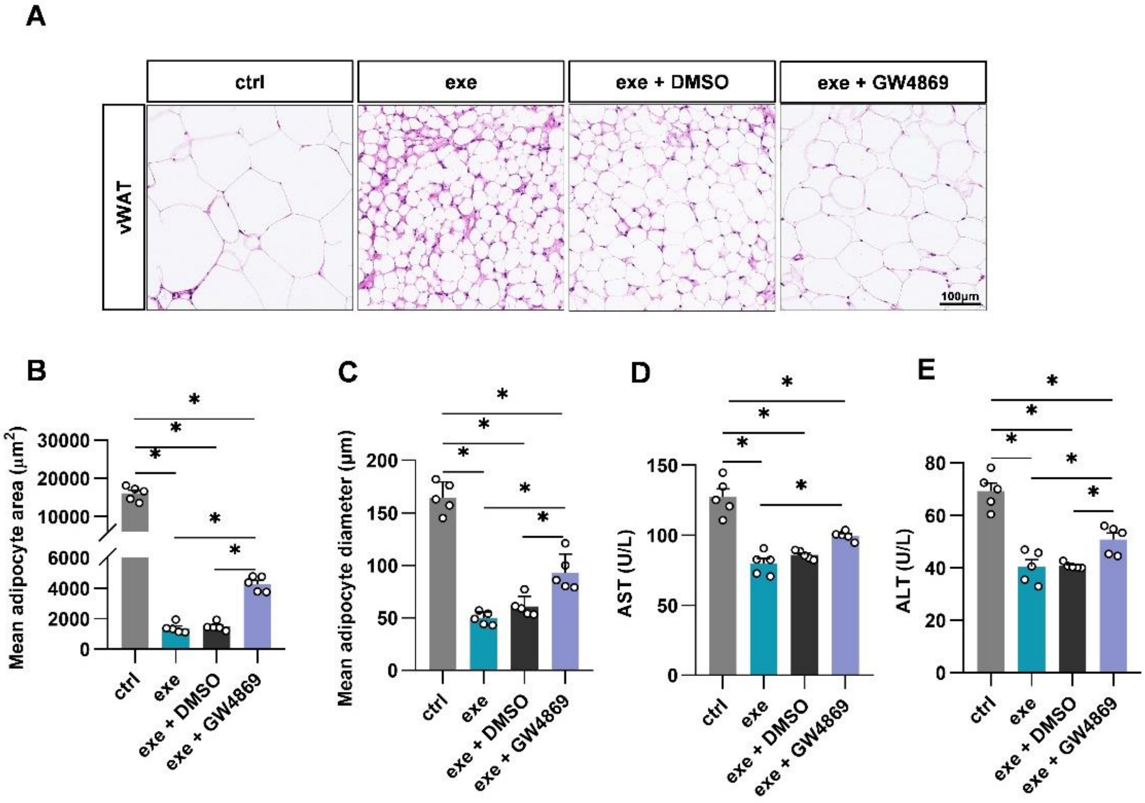

Supplementary Figure 4. GW4869 inhibits the benefits of exercise in reducing adipocyte size and improving hepatic function in ApoE$^{-/-}$ mice. A. HE staining of vWAT from mice with indicated treatments. B. Quantification of adipocyte areas. C. Quantification of adipocyte diameters. D-E. Serum levels of AST (D) and ALT (E) in mice from each group. Data are presented as mean ± SEM. *, $P<0.05$; kruskal-Wallis test with Dunn's post hoc test. n = 5 per group. ALT, Alanine aminotransferase; AST, Aspartate aminotransferase.

© 2022. Wang Y et al. Published online at http://www.aginganddisease.org/EN/10.14336/AD.2022.1131

SUPPLEMENTARY DATA

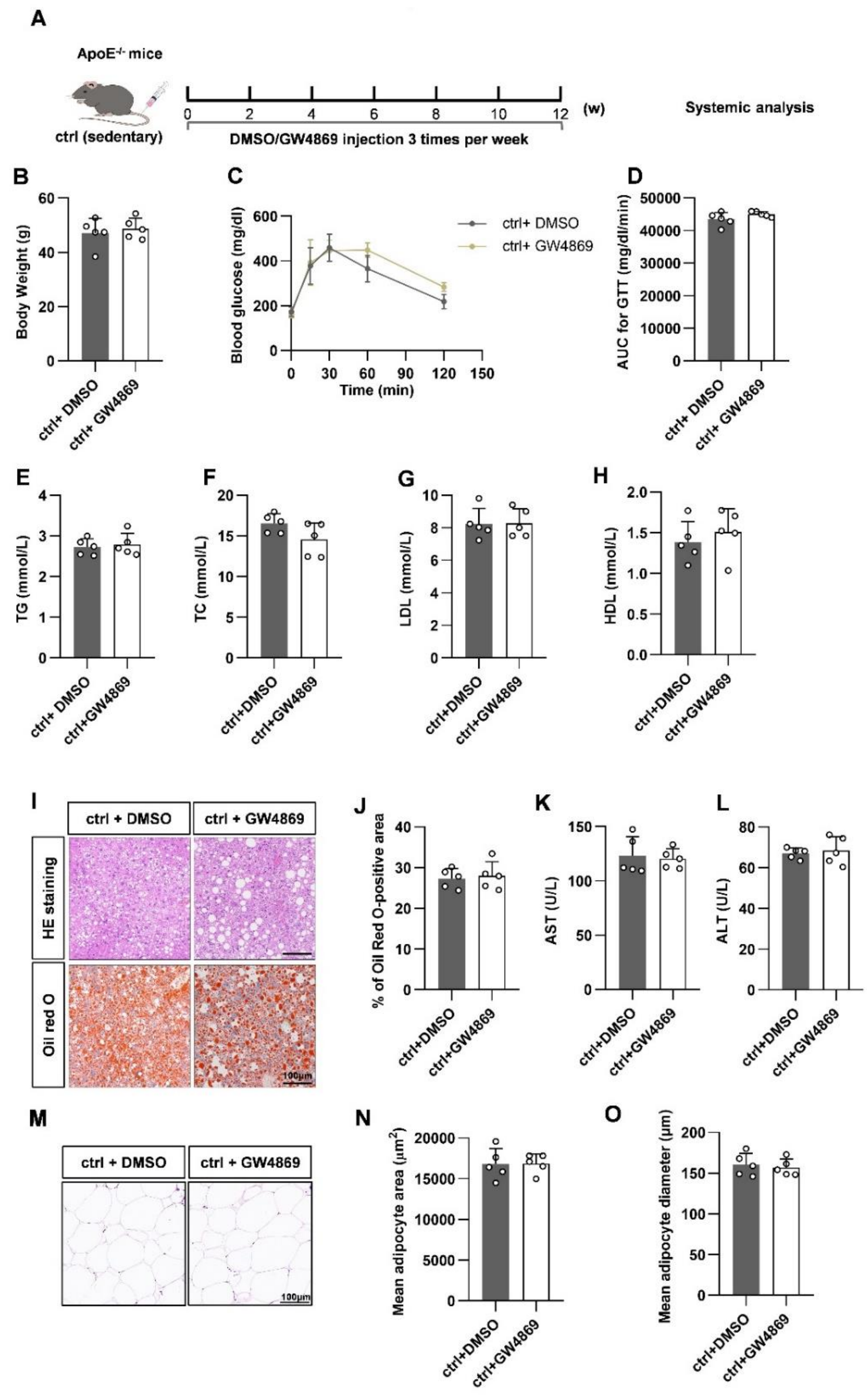

Supplementary Figure 5. GW4869 has no effects on metabolic profile in non-exercised ApoE$^{-/-}$ mice. A. Illustration of animal grouping and experimental procedure. B. Body weights of the mice with indicated treatments. C. Intraperitoneal glucose tolerance test in mice with indicated treatments. D. AUC of the GTT performed in mice with indicated treatments. E-H. Serum lipid levels from indicated groups. I. HE staining (top) and Oil red O staining (bottom) of liver sections from indicated groups. J. Percentage of Oil Red O positive area in livers from indicated groups. K-L. Serum levels of AST (K) and ALT (L) in mice from each group. M. Representative HE staining of vWAT from indicated mice. N. Quantification of mean adipocyte area of vWAT. O. Quantification of mean adipocyte diameters of vWAT. Data are presented as mean ± SEM. *, P<0.05; two-tailed Mann-Whitney U test. n = 5 per group.

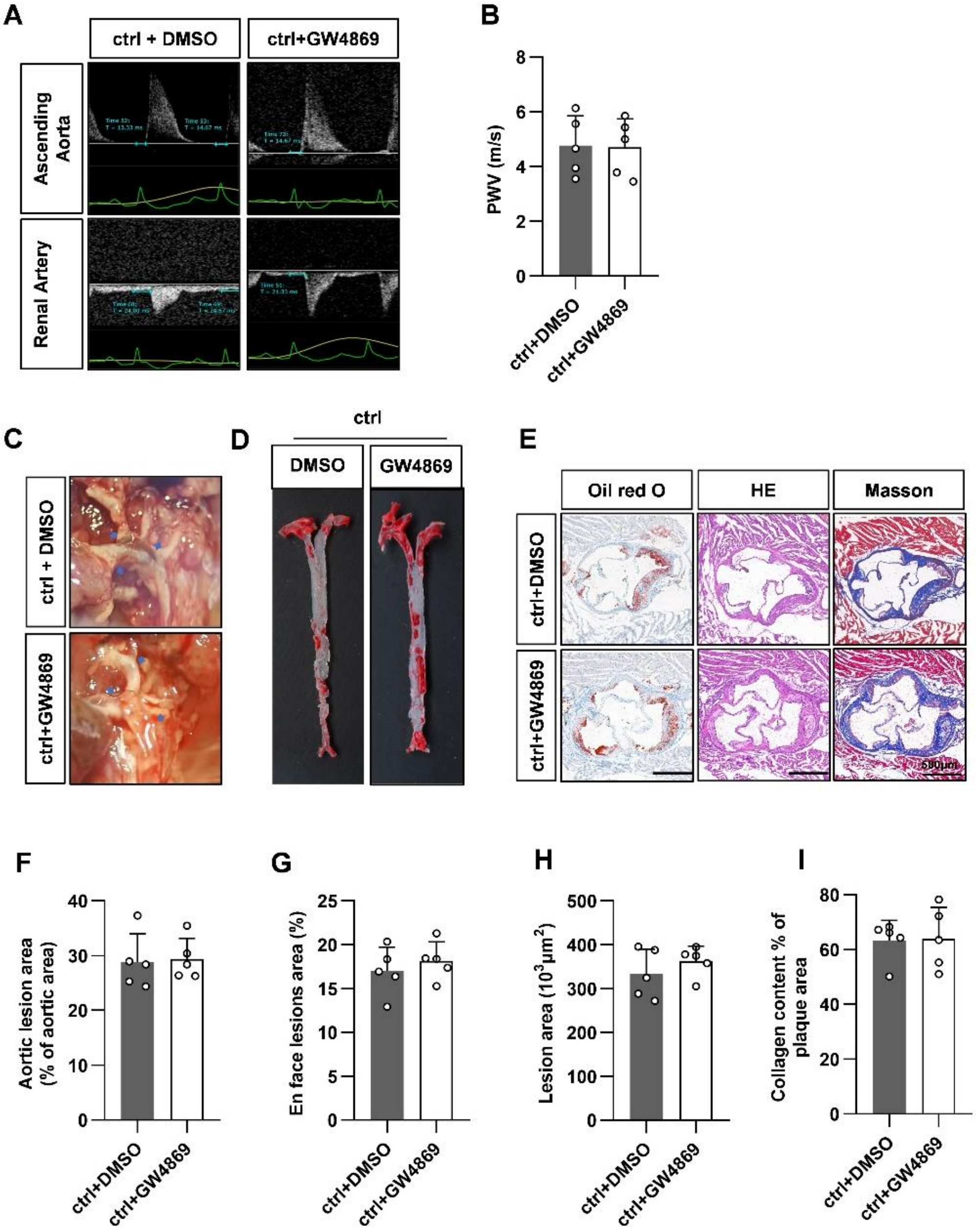

Supplementary Figure 6. GW4869 has no effects on atherosclerosis progression in non-exercised ApoE$^{-/-}$ mice. A. Representative images showing the analysis of PWV. B. PWV in ApoE$^{-/-}$ mice treated as indicated. C. Representative aortic arch view of the atherosclerotic lesions in ApoE$^{-/-}$ mice treated as indicated. D. Oil red O staining of the aortic tree in ApoE$^{-/-}$ mice treated as indicated. E. Representative images of the cross-sectional view of the aortic roots stained with Oil red O, HE and Masson. F. Percentage of the atherosclerotic area in the aortic arch. G. Percentage analysis of the atherosclerotic region from D. H-I. Percentage analysis of the atherosclerotic region from E. Data are presented as mean ± SEM. *, $P<0.05$; two-tailed Mann-Whitney U test. n = 5 per group.

© 2022. Wang Y et al. Published online at http://www.aginganddisease.org/EN/10.14336/AD.2022.1131

SUPPLEMENTARY DATA

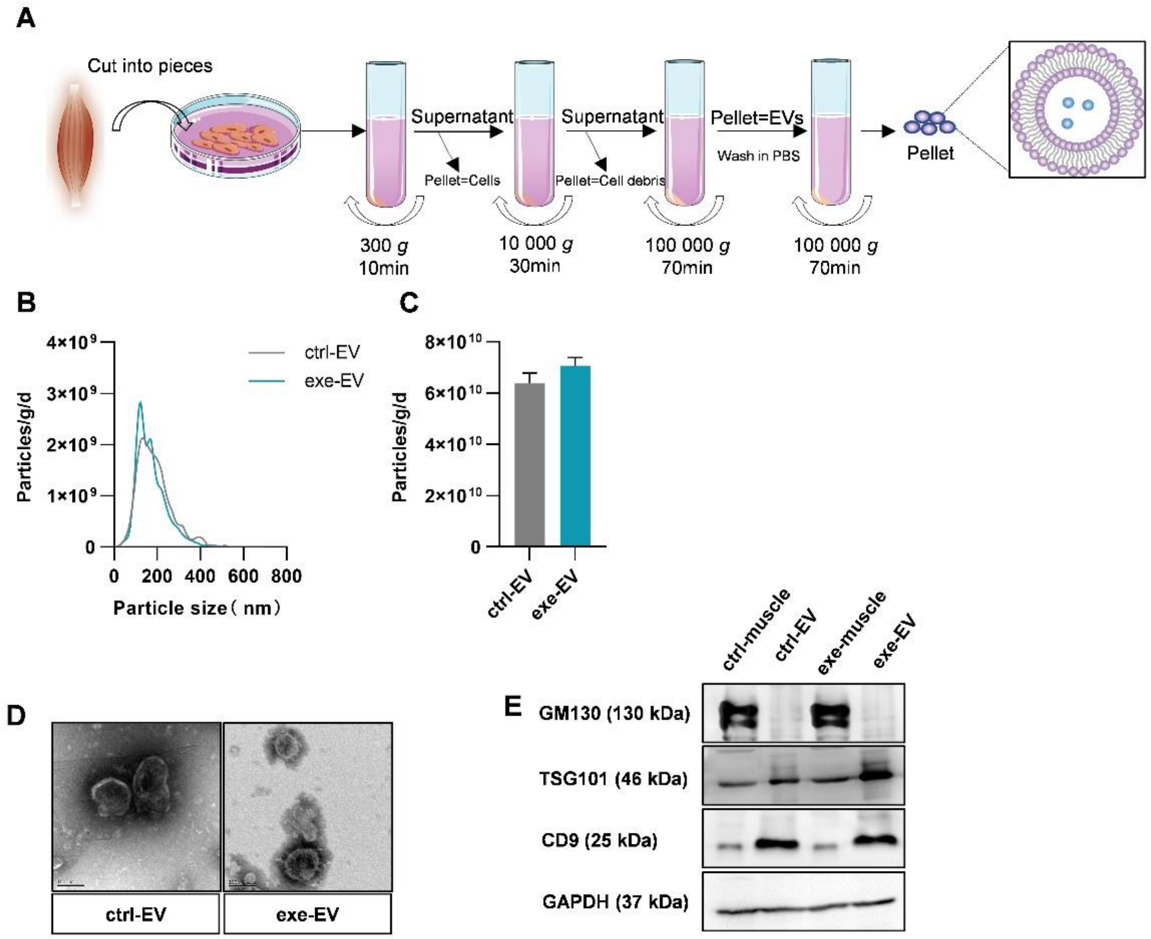

Supplementary Figure 7. Preparation and characterization of skeletal muscle-derived EVs. A. Illustration of the procedure of EV isolation. B. Representative nanoparticle tracking analysis demonstrating the size distribution of EVs purified from the muscle of control and exercised mice. C. EV concentration from the muscle of control and exercised mice. Data are presented as mean ± SEM. Two-tailed Mann-Whitney U test. n = 5 per group. D. Representative transmission electron microscope (TEM) images of EVs from the muscle of control (sed-EV) and exercised mice (exe-EV). E. Western blot analysis of exosomal inclusive and exclusive markers in muscle and derived EVs from muscle of sedentary and exercised mice. EVs, extracellular vesicles; ctrl-EV, extracellular vesicles derived from the muscle of control mice; exe-EV, extracellular vesicles derived from the muscle of exercise mice.

© 2022. Wang Y et al. Published online at http://www.aginganddisease.org/EN/10.14336/AD.2022.1131

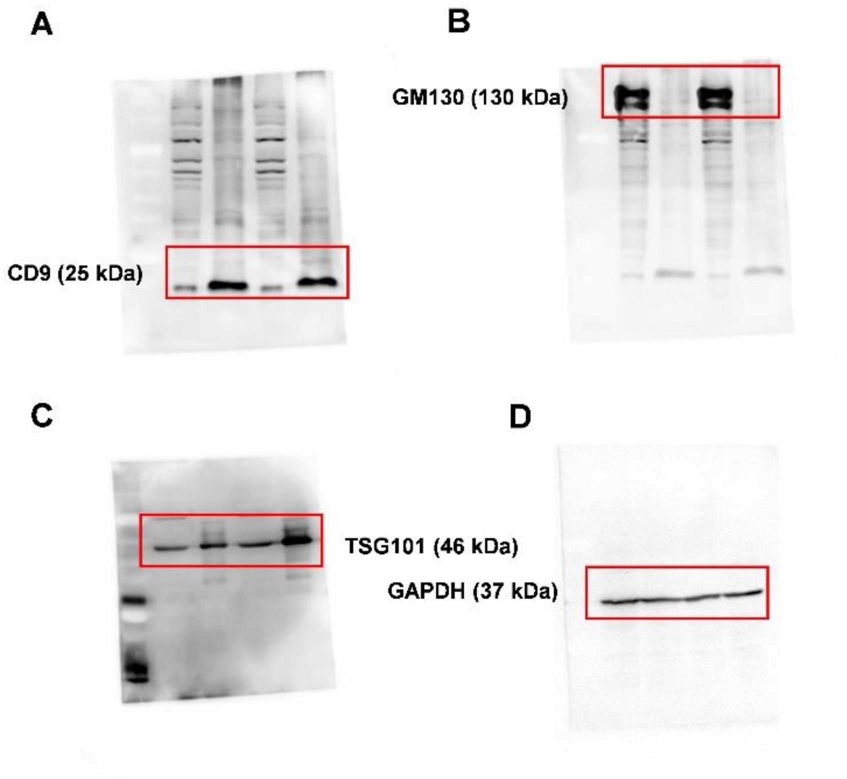

Supplementary Figure 8. Uncropped Western blot data corresponding to Figure S7E.

© 2022. Wang Y et al. Published online at http://www.aginganddisease.org/EN/10.14336/AD.2022.1131

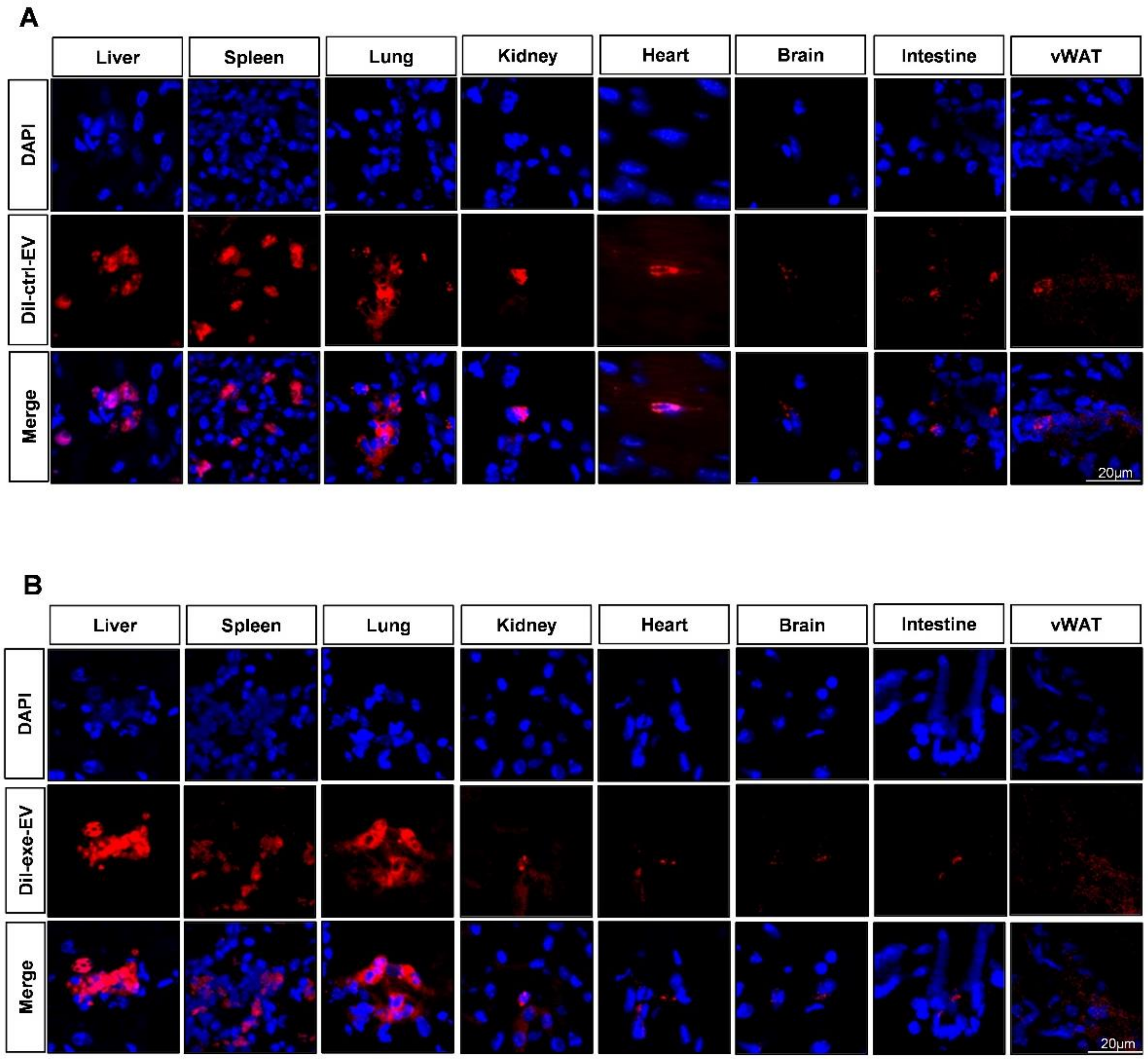

Supplementary Figure 9. Distribution of EVs in different organs of WT mice. A-B. Representative confocal images of DiI-labeled ctrl-EV (A) and DiI-labeled exe-EV (B) in different organs. n=5 per group.

© 2022. Wang Y et al. Published online at http://www.aginganddisease.org/EN/10.14336/AD.2022.1131

SUPPLEMENTARY DATA

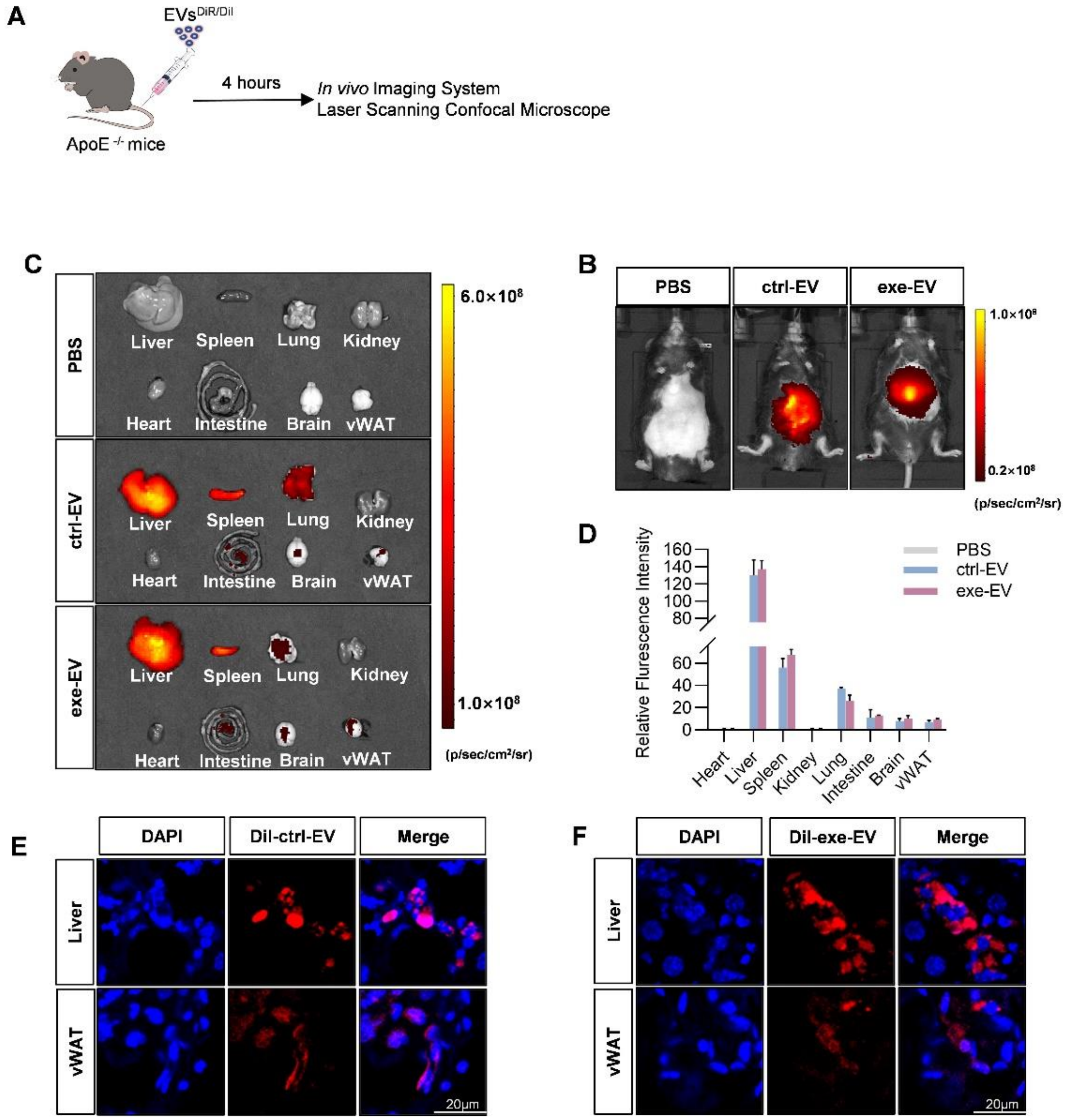

Supplementary Figure 10. Distribution of EVs in ApoE$^{-/-}$ mice. A. Illustration of experimental procedure. B. Representative images of the distribution of DiR-labeled EVs analyzed by *in vivo* imaging system. EVs were labeled with the fluorescent dye DiR and injected into mice via intravenous injection. C. *Ex vivo* fluorescence imaging analysis of the distribution of the DiR-labeled EVs in different organs. D. Quantification of fluorescence Intensity in B. E-F. Representative confocal images of the localization of DiI-labeled exosomes in liver and vWAT. n=5 per group.

© 2022. Wang Y et al. Published online at http://www.aginganddisease.org/EN/10.14336/AD.2022.1131

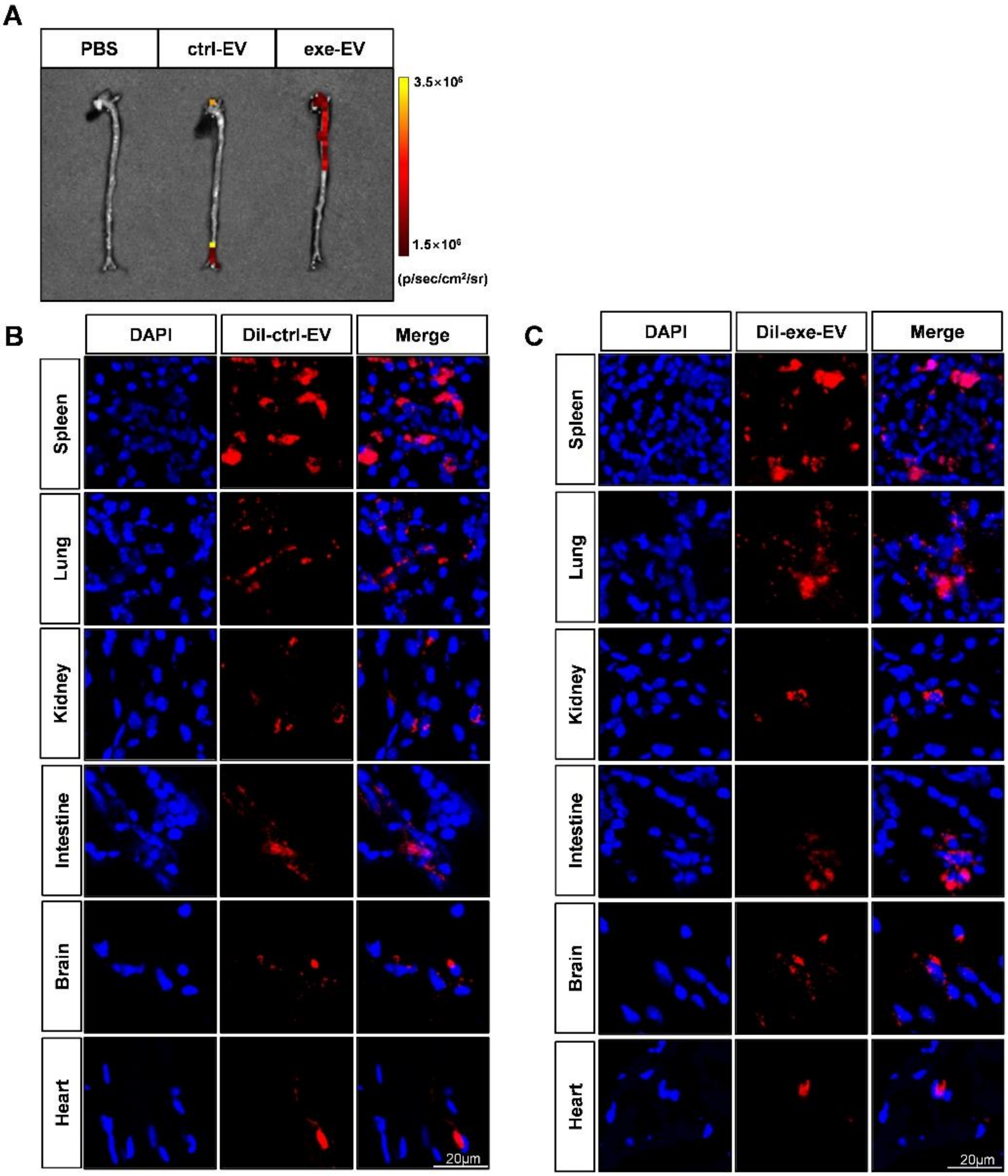

Supplementary Figure 11. Distribution of EVs in aorta and some other organs of ApoE$^{-/-}$ mice. A. Representative images of *Ex vivo* fluorescence imaging of the DiR-labeled EVs in the aorta. B-C. Representative confocal images of DiI-labeled ctrl-EV (B) and exe-EV (C) in indicated organs. n=5 per group.

© 2022. Wang Y et al. Published online at http://www.aginganddisease.org/EN/10.14336/AD.2022.1131

SUPPLEMENTARY DATA

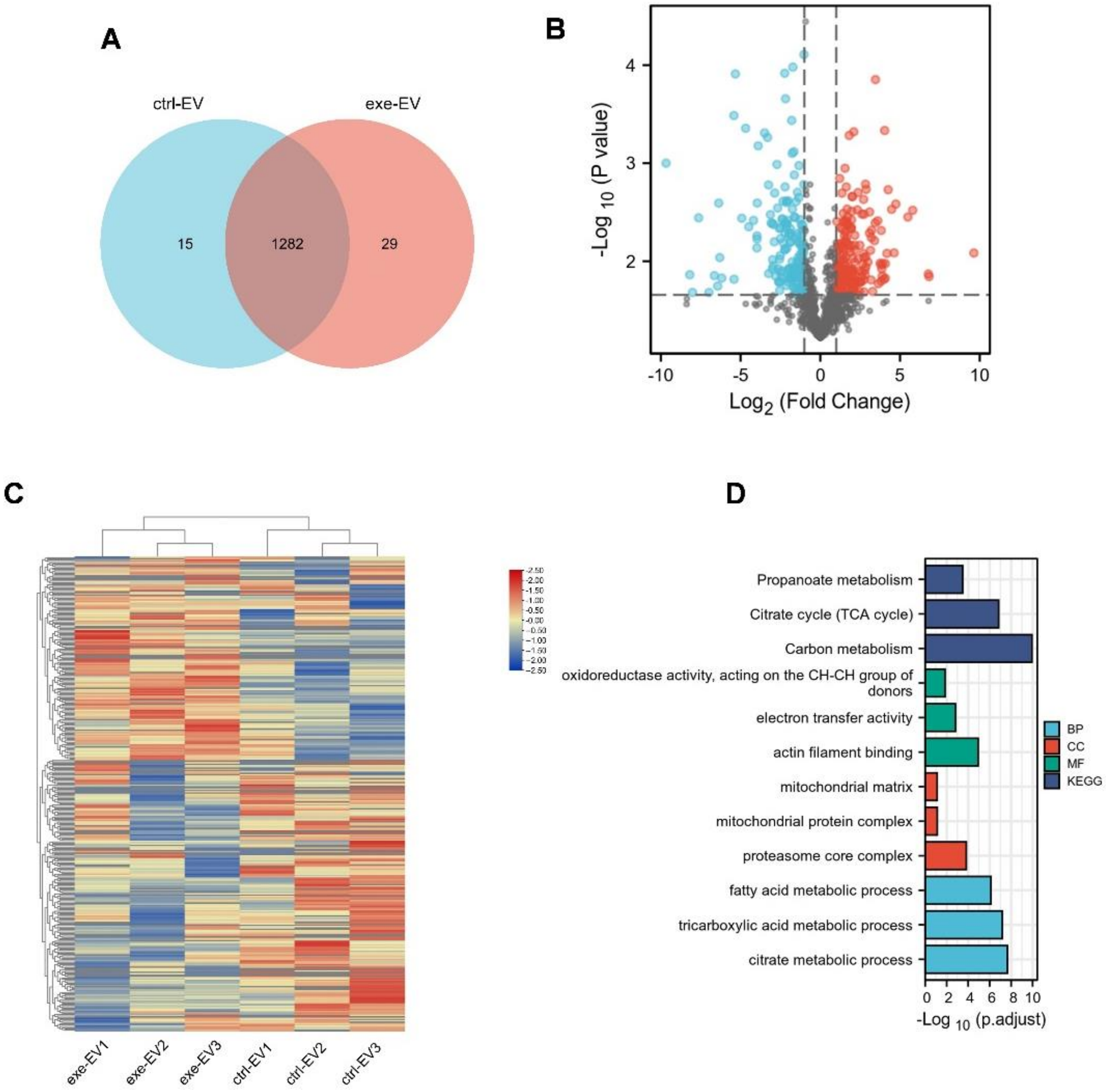

Supplementary Figure 12. Proteomic profiling of EV components from control and exercised skeletal muscle. A. Venn diagram of proteins identified in ctrl-EV and exe-EV (n=3). B. Volcano plot showing the protein difference between ctrl-EV and exe-EV. C. Cluster analysis of differential proteins between ctrl-EV and exe-EV (n=3). D. Pathway enrichment analyses of proteins up-expressed in exe-EV. BP, biological process; CC, cellular component; MF, molecular function.

© 2022. Wang Y et al. Published online at http://www.aginganddisease.org/EN/10.14336/AD.2022.1131

SUPPLEMENTARY DATA

Supplementary Table 1. The leading candidate pathways for proteins significantly up-regulated in exe-EVs.

KEGG Pathways	p-value	Corrected p-value	Genes
**Carbon metabolism** mmu01200	9.1448E-13	1.1522E-10	Pfkl/Pgk2/Suclg1/Acss2/Pgls/Sdha/Sdhb/Echs1/Acat1/Idh3b/Dlat
**Citrate cycle (TCA cycle)** mmu00020	1.1447E-09	1.4424E-07	Suclg1/Sdha/Sdhb/Acly/Idh3b/Dlat
**Propanoate metabolism** mmu00640	2.5733E-06	3.2404E-04	Suclg1/Acss2/Echs1/Acaca/Acat1
**Fatty acid metabolism** mmu01212	5.1946E-05	6.5503E-03	Acadl/Fasn/Echs1/Acaca/Acat1
**Pyruvate metabolism** mmu00620	1.2034E-04	4.6747E-02	Acss2/Acaca/Acat1/Dlat

© 2022. Wang Y et al. Published online at http://www.aginganddisease.org/EN/10.14336/AD.2022.1131
